# The Role of Demand Factors in Utilization of Professional Care during Childbirth: Perspectives from Yemen

**DOI:** 10.5402/2011/382487

**Published:** 2011-09-18

**Authors:** Annica Kempe, Fatoom Noor-Aldin Alwazer, Töres Theorell

**Affiliations:** ^1^National Prevention of Suicide and Mental Ill-Health, Department of Public Health Sciences, Karolinska Institutet, 171 77 Stockholm, Sweden; ^2^National Association of Yemeni Midwives, Al-Qiyadah Street, Al-Bait Assaeed Furniture Building, Third Floor, Flat 15, Sana'a, Yemen; ^3^Population Sector, Deputy Office, Al Hasaba, Ministry of Public Health and Population, Sana'a, Yemen; ^4^Stress Research Institute, Stockholm University, 106 91 Stockholm, Sweden

## Abstract

*Background*. Utilization of professional care during childbirth by women in low-income countries is important for the progress towards MDG 5. In Yemen, home births have decreased minimally during the past decades. *Objective*. The study investigates the influence of socio-demographic, birth outcome and demand factors on women's future preference of a home or institutional childbirth. *Method*. We interviewed 220 women with childbirth experience in urban/rural Yemen. We performed bivariate chi-square tests and multiple logistic regression analysis. A multistage sampling process was used. *Results*. The issues of own choice, birth support and birth complications were the most important for women's preference of future location of childbirth. Women who had previously been able to follow their own individual choice regarding birth attendance and/or location of childbirth were six times more likely to plan a future childbirth in the same location and women who received birth support four times more likely. Birth complications were associated with a 2.5-fold decrease in likelihood. *Conclusions*. To offer women with institutional childbirth access to birth support is crucial in attracting women to professional care during childbirth. Yemeni women's low utilization of modern delivery care should be seen in the context of women's low autonomy and status.

## 1. Introduction

Maternal mortality is increasingly being recognized as a violation to women's right to survive pregnancy and childbirth. Recent research shows that more than 343,000 women die during childbirth or due to pregnancy-related causes every year [1]. Underutilization of maternal and child health (MCH) services has been identified as a major factor contributing to the high maternal mortality rates (MMR) in low-income countries. Knowledge about factors affecting the use of institutional birth is important to eliminate barriers and attract women to modern care. Skilled birth attendance, first focused during the Safe Motherhood (SMH) meeting in Sri Lanka in 1997 [2], has been at the center of international effort to improve maternal and child health during the past decades. Indicators for monitoring progress of the Millennium Development Goals (MDGs) relating to reproductive health and rights are (a) the MMR and (b) proportion of births attended by skilled health personnel. 

The impact of female education on the use of MCH services is well known through previous research [3–7] and has been shown to lead to the acceptance of modern medical practices [8–10]. However, it has been noted that about one-half of the very great educational differentials in utilization found across low-income countries can probably be explained by economic advantages associated with education [8, 11]. Research from different parts of the world has shown the positive consequences of urban residence, such as access to better health facilities, infrastructure, information, and knowledge, all of which are often lacking in rural areas [12, 13]. Higher utilization by educated women may thus simply reflect the effects of residence as service availability and educational opportunities are concentrated in urban areas. Increasing evidence from low-income countries suggests that cultural and “demand factors” play an important role in the underutilization of MCH services [14–20]. Factors related to women's autonomy have been found to have a substantial influence on utilization in some settings [21–24]. Closely connected to this is the exercising of own authority in the context of traditional childbirth [25, 26], allowing women status within the realm of childbearing and the recognition of childbirth as a community as well as a biological event. A review study from 2009 on the determinants of service use during childbirth [27] emphasized the importance of considering as many influencing factors as possible in any analysis of service use.

The maternal mortality ratios in Yemen are estimated at 430 maternal deaths per 100,000 live births [28]. The high level of maternal mortality reflects the low value placed on women's life by society and also by women themselves [29]. In spite of the high risk of death or illness during childbirth, many women endure frequent pregnancies in order to gain the attention and approval of their husbands and in-laws. The expansion of MCH services within the Safe Motherhood Initiative (SMI) in Yemen has not been found to relate to women's use of the services [30]. Home births have decreased by only two percent during the last decade and are now estimated at 76% of the total number of births [28]. According to the 2003 household-based Family Health Survey [30] nearly 64% of the total number of maternal deaths occurred at home. A study in Yemen [31] from 1993 found that the final and strongest risk factor for maternal mortality was seriousness of condition at admission to the hospital. A high proportion of women delayed seeking medical care at an appropriate time after signals of complications arose at home. The government has committed itself to the Safe Motherhood Strategy and more recently to the MDGs aiming at reducing the MMR by three-quarters by the year 2015. Based on experience from a number of countries in the region, a code of ethics for midwives was recently developed by the Yemeni Midwives Association. Highlighting factors within MCH care which would increase women's utilization of the services was an important aspect of this work. Data obtained within the present study was used here to illustrate women's own conceptions of quality of care and demand factors.

### 1.1. Aim of the Study

The aim of the study was to examine the influence of socio-demographic, delivery outcome, and demand factors on women's preferred location of childbirth in case of a future pregnancy, seen against the background of the previous childbirth. 

## 2. Material and Methods

### 2.1. Participants

Participants in the study were women with childbirth experience in selected areas of Yemen: Aden (Aden Governorate), Lahej (Lahej Governorate), Seyoon (Hadramout Governorate), Taiz (Taiz Governorate), and Zabid (Hodeidah Governorate). The study constitutes a substudy to a comprehensive study of the midnineties concerning the quality of maternal and neonatal healthcare services seen through women's eyes [32] conducted on behalf of Save the Children Sweden (SCS). Districts were sampled with the ambition of representing the widest possible range of geographical, cultural, ethnic, and infra-structural characteristics. A multistage (stratified-purposive-random) sampling process was used. Initially, a pilot study was conducted in rural/urban Taiz. One rural and one urban stratum in each of the five target districts were then selected. Secondly, (a) an area in the immediate vicinity (up to half an hour walking distance) from the local SCS MCH clinic and (b) an area of at least two hours of walking from the same clinic, were purposely selected. Thirdly, a random selection of households for the interviews with women with childbirth experience was made. Interviews were conducted with the female head of each identified household. When approached for the interview, women were told about the purpose of the study and asked whether they would give their consent to participate. All women agreed to participate.

### 2.2. Procedures

Study participants were interviewed by means of interview-administrated questionnaires containing close- and open-ended questions. A quota of 22 interviews was allocated to each of the clusters. Thus the target number of interviews totaled 220. The questionnaire was translated from English into Arabic. Interviews were made by four national research assistants with a long experience from MCH-related work in rural/urban Yemen and one Sudanese research assistant formerly employed in MCH work in Yemen. Interviews were conducted according to the preferred pace of the interviewee and lasted for 1–3 hours. Special care was taken not to interfere with the heavy household burdens of women, which meant that interviews were sometimes interrupted for cooking, attending to animals, or hanging wash to dry. All women were interviewed alone and in a few cases this meant that interviewers had to return a second time in order to secure privacy from husbands and in-laws. All performed interviews were discussed on the same evening among the members of the research team in order to check for any lack of clarity.

### 2.3. Variables and Questions Posed

Institutional births were defined as those occurring in a hospital or in a clinic/health unit. Women's previous location of childbirth was addressed through the question: “Where did you deliver your child? Home/clinic or health unit/hospital.” Prospective health care seeking in case of a future pregnancy was addressed through the question: “If you had another baby, would you choose the same place of delivery? Yes/No.”

A large number of variables were selected to illustrate the social and demographic background of the woman respondent: age at the time of the most recent childbirth, place of residence, number of dependents, dead children, women's literacy and educational background, husband's educational background, women's employment with a wage salary during the last three months, occupation of husband, other sources of family income, and distance by walking to the location of childbirth. 

Variables related to childbirth included both birth complications and “demand” factors related to the childbirth experience. The occurrence of birth complications was addressed through the question: “Did you suffer any birth complications? Yes/No.” Further detailed information given by women about these complications is not used in this study. Potential “demand factors” were discussed in the research group during the pretesting of the questionnaire. It was clear that some aspects of childbirth were central to women's positive experience and evaluation: whether women were able to execute and maintain authority during childbirth (“Did you feel that you were the authority at birth? Yes/No”), whether birth support from one or more additional person(s) besides the attending staff was allowed (“Were you allowed to have with you the people of your choice? Yes/No”), and whether women were able to remain in close contact with the infant directly following birth (“Where was the child put immediately within the first few minutes after birth? Skin-to-skin, wrapped/dressed in arms, separate bed/elsewhere near, other place in room but out of contact, separate room/place out of sight”). Whether women had been able to make a personal choice of birth attendance/location of childbirth seemed important in the context of demands (“Did you choose your midwife/your place of delivery yourself? Yes/No”).

### 2.4. Statistical Analysis

Descriptive statistics were used to identify variables associated with women's previous and preferred future location of childbirth. Social and demographic variables, delivery outcome variables, and “demand” variables were examined. The data was analyzed in the statistical programs StatView SE Graphics and JMP. 

The dependent variable for the study of the previous childbirth is trinary and for that concerning choice of future location of childbirth, binary. The three alternatives in the study of the previous childbirth are (1) previous childbirth took place at home, (2) previous childbirth took place in a clinic or health unit, and (3) previous childbirth took place in a hospital. For the study concerning preference of future location of childbirth, the two alternatives are (1) future childbirth is planned in the same location and (2) future childbirth is planned in a different location. Separate chi-square tests were conducted to first determine which of the possible explanatory variables were significantly associated with (1) women's previous location of childbirth and (2) women's preference of same or different location in case of a future pregnancy. 

In order to identify the variables independently associated with both the previous and the preferred future location, multiple logistic regression analysis was conducted. The rule was to include only variables that showed significant association with the previous or preferred location of childbirth. Two variables, “own choice of birth attendance/location of childbirth” and “location of previous childbirth,” were strongly related. To minimize the number of variables, a choice was made to include only that of “own choice” in the multiple logistic regression analysis. One variable, “mother-infant bonding,” was collapsed into a dichotomous variable, since the number of subjects in the first and last categories were very small. Once the final models were selected, odds ratios for each significant explanatory variable were calculated and interpreted to determine how the odds of a previous birth at home or in an institution/a planned birth in the same location of childbirth increases or decreases given the presence (or absence) of a particular explanatory variable. The program (“Fit Model”, JMP) estimates the effect on the y variable of a change of one step in the explanatory variable after all other variables have been accounted for. 

## 3. Results

### 3.1. Previous Childbirth

Of the 220 women participating in the study, 151 (69%) gave birth at home and 69 (31%) gave birth in an institution. Most of the institutional births (55 out of 69) took place in a hospital and less than a fifth [14] in a clinic or health unit. Variables analyzed in relation to women's previous childbirth are shown in Table 1.

Table 2 shows the results of the multiple logistic regression analysis. Of the 13 variables which were included in the analysis, six variables showed an independent statistically significant association with women's previous location of childbirth: “Women's authority during childbirth,” “birth support,” “own choice of birth attendance/location of childbirth,” “antenatal care,” “family income from land/cattle,” and “distance by walking to the location of childbirth.” If a woman had had a birth support person present there was a 36% increase and if she had remained in authority a 26% increase in likelihood that she would have given birth at home. Own choice of birth attendance/location of childbirth increased the likelihood of a home delivery by 14%, family income from land or cattle by 14% and the distance by walking to the location of delivery by 47%. Antenatal care decreased the likelihood of a home birth by 11%.

### 3.2. Future Childbirth

Five women in the study population stated that they were not going to be pregnant again due to older age or being a widow with no intention to remarry. These women were excluded from the analysis. Of the 215 women remaining in the study, 186 (87%) stated that they would give birth again in the same location in case of a future pregnancy while 29 (13%) were reluctant to do so. Variables investigated in relation to women's preference of same or different future location of childbirth are shown in Table 3.

In the bivariate analysis, significant relationships were found with a number of variables intimately related with the childbirth experience and nonsignificant relationships were found with all social and demographic variables except one: distance by walking to the location of childbirth. Whether women experienced childbirth at home or in an institution strongly influenced the choice of prospective location. Of the 148 women with experience of giving birth at home, 140 (95%) stated that they would choose a home birth again while only 46 (69%) of the 67 women who gave birth in an institution would choose the same location again. In the group of women with experience of institutional childbirth, future preference depended to a large degree on whether childbirth took place in a clinic/health unit or in a hospital: 13 out of 14 (93%) women who experienced childbirth in a clinic or a health unit would return to the same location while only 33 out of 53 (62%) women with experience of childbirth in a hospital expressed a wish to return.

#### 3.2.1. Same or Different Future Location of Childbirth: A Matter of Women's Past “Own Choice”, Birth Support and Birth Complications

**Figure 1:**
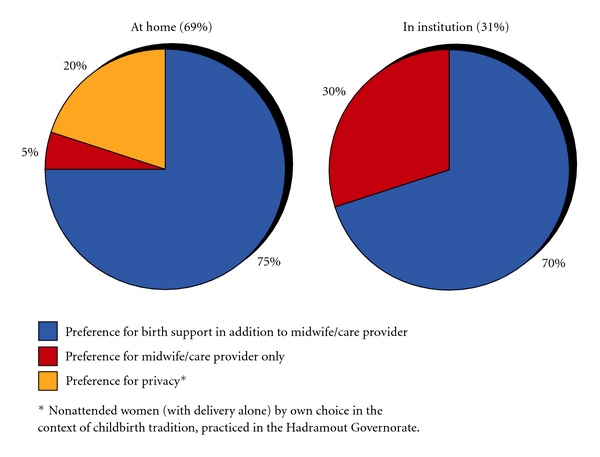
Percentage of women with preference for support versus privacy during childbirth in the home and institutional sector (*n*: 220).

Similarly as in the baseline study, to explore which variables were independently associated with women's choice of future childbirth location, we performed a multiple logistic regression analysis (Table 4). As a result of the variable selection procedure, six variables remained in the multiple logistic regression analysis. “Own choice of birth attendance/location of childbirth,” “birth support,” and “birth complications” were the only three variables that showed an independent statistically significant association with women's preferred future location of childbirth. Women who had been able to follow through on their own choice of birth attendance and/or location of childbirth previously had a 6-fold and women who had received birth support from an additional person other than the midwife/care provider a 4-fold possibility of planning childbirth in the same location. By contrast, women who reported birth complications had a 2.5-fold lower probability of wanting to give birth again in the same location than women on average in the study population. The percentage of women with preference for a birth support person, midwife/careprovider only or for privacy during childbirth at home and in an institution is shown in Figure 1.

#### 3.2.2. “Own Choice,” a Strong Concern to Yemeni Women in Matters of Childbirth

What characterized the women who would choose a different location of childbirth in the home and institutional group respectively? In an investigation of the two groups separately (not presented in full here), bivariate analysis showed that the factor of previous “own choice” was the predominant factor for the wish to change location both among the women with experience of home birth (*P* = .018, DF: 1, total chi-square: 5.629, *n*: 8) and among the women with experience of institutional childbirth (*P* = .0004, DF: 1, total chi-square: 12.474, *n*: 21). Among women with experience of home birth, “women's authority” was the only additional variable showing a significant (positive) association with women's preference of same location while among the women with experience of institutional childbirth, “birth complications” was the only additional variable showing a significant (negative) association with same location. Only 14 women gave birth in a clinic or health unit, making comparisons between clinic and hospital births difficult. Analysis of women who gave birth in hospital only (*n*: 55) showed that the variables of “own choice” and “birth complications” again emerged in multiple regression analysis as those with an independent statistical association to women's preference for same or different location. More than 75% of the women with experience of hospital birth who previously chose this location of childbirth looked positively upon a future hospital birth.

### 3.3. Impact of a Current Pregnancy on Women's Choice of Future Location of Childbirth

The time for provision of information from women was the period since the most recent childbirth, which in some cases was several years ago. In order to know whether women who were currently pregnant had different preferences for a prospective location of childbirth than nonpregnant women, women in a current pregnancy (*n*: 44, 20%) were compared with nonpregnant women using a bivariate chi-square test. No difference between the groups was shown.

## 4. Discussion

Maternal mortality remains high in Yemen, but in spite of the fact that skilled attendance during childbirth is key to improved outcomes, women underutilize modern care. This study of Yemeni women with childbirth experience shows that women's own choice of birth attendance and/or location has a strong bearing on the fact that home births still constitute 76% of the total number of births. Women's view of childbirth as an event supported by other women participating in the birth was the overriding explanation for this preference. Social and demographic factors were of less importance for women's preference of location of childbirth in case of a future pregnancy. Neither did a current pregnancy influence women's preference. 

What characterized the women who wanted to change location of childbirth? Most women who preferred a different location of childbirth in the future were women who experienced birth complications within the frame of institutional care and wanted a future home birth. These findings relate closely to the findings from a previous study [31], which investigated birth complications in hospitals in former North Yemen. The study showed that women delayed seeking medical care until complications had already occurred at home, reflecting the reluctance of women to plan for an institutional childbirth in the first place, thus adding to the severity of complications in institutions. Women's unwillingness to plan for a future institutional childbirth could probably be partly explained by the trauma resulting from this experience. In 2008, Women's National Committee (WNC), the main women's organization in Yemen, explained the persistent low utilization of health services during pregnancy and childbirth by the limited and/or poor quality of these services, particularly in the rural areas, the maltreatment of women at services centers, in addition to the transportation costs to such centers [33]. Quality issues as seen by women were again emphasized in the ethical code for midwives recently produced.

Few studies have looked at factors related to women's autonomy and perceived need of care during childbirth. Respondents' emphasis on support during childbirth, a strong element in traditional care in Yemen [32], indicates that women's low status in the society relates closely to the function of the traditional childbirth sector, which allows for the support and solidarity among women on the community level. Interestingly, it also plays a strong part in women's inability to follow through on an individual choice of an institutional childbirth, prevented sometimes by the husband or other male relatives, a situation that was also recently highlighted by the WNC [33]. That the issue of making a personal choice regarding birth attendance, birth location, or both was of crucial importance both for home birth women who wanted a future institutional childbirth and among institutional birth women who wanted a future home birth shows the importance of own choice for the Yemeni woman and should be seen in the context of women's low autonomy and status in the society as a whole. The question posed to women concerning “own choice” concerns birth attendance, location of childbirth, or a combination, and it is not possible within the scope of this study to know to what extent women's choice of birth attendance was determining the choice of location. The possibility of choosing birth attendance is not evenly distributed in the home and institutional sector. The emphasis in this study, however, is on women's own perception and statement of having made a personal choice per se in the childbirth situation. A closely related concept, the exercising among Yemeni women of own authority during childbirth, has been explored in a previous study [34]. 

The stated preference of birth support of particular women who experienced birth complications, for the most part reluctant to plan a next institutional childbirth in case of a future pregnancy, is an important finding in this study. The impact of women's fear of the solitude of modern delivery practice on this reluctance is strong, particularly in the rural areas (unpublished observation). The value of birth support has been documented in research from around the world [35–37]. Information from a WHO Cochrane Review [38] that looked at 16 studies of over 13,000 women has clearly shown that a woman, supported during her labor and birth, has a more positive experience and fewer labor complications. Supported women have higher rates of spontaneous vaginal deliveries, lower rates of vacuum deliveries and cesarean sections, less need for oxytocin augmentation, and shorter labors.

The situation that home births still constitute 76% of the total number of births in Yemen, a country with one of the lowest ratios of primary school attendance among girls in the world [39], serves to illustrate previous findings [4–7] that the educational level of women and girls is important for the choice of location of childbirth. However, it also illustrates women's own demand of a community-based, “humanistic” quality of care valued by them beyond, and in spite of, the challenges of current Safe Motherhood strategies. In our analytical model, the variable of “own choice” has a central position. It could be argued that human beings always tend to choose conditions that they are familiar with when planning the future. However, the crucial question is the following: how can women in Yemen and similar countries be convinced that they would benefit from modern care during childbirth? Factors unrelated to safety seem to be important in future choice. When modern care incorporates the needs and priorities that women have, future attendance to modern delivery is likely to increase. Closer cooperation between modern and traditional childbirth sectors is therefore crucial for women in transition from traditional to modern care. It has been shown in a study from Peru [14] that cultural adaptation of birthing services can manage to increase the proportion of births in a health facility from 6 to 83% percent over a period of eight years only. The inclusion of family and TBAs in the health facility as well as the integration of other traditional elements as the use of rope and bench for vertical delivery position was responsible for this change. In the present study, the relative satisfaction and willingness to return again for a future childbirth expressed by women with childbirth experience from clinics or health units, even though only a small number of births were studied, indicate that women will give birth in institutions if their needs are met. These women to a considerable degree perceived themselves to be in authority during the birth and were able to enjoy the presence of a birth support person in a fair number of cases. 

The strong call for a closer cooperation between the modern and traditional childbirth sectors, which findings of this study support, is imperative in order to attract women in settings with a high maternal mortality and morbidity to professional care during childbirth. 

### 4.1. Strengths and Limitations

The study has taken its data directly from women, the most important beneficiaries of both MCH and PHC services but nevertheless rarely heard. To our knowledge, few studies have investigated the influence of demand factors in relation to social, demographic, and birth outcome variables on women's utilization of professional care during childbirth. The fact that interviews were taking place in the homes of women rather than elsewhere meant that each woman was interviewed in a setting where she felt the most at ease. Talking to each woman alone was important as, in some cases, women had been influenced by older relatives or husbands to give birth either at home or in an institution. All women participated in the study. The selection of participants was stratified in such a way that representativeness was ensured. Interviews were conducted by midwives and medical doctors carefully selected on the basis of educational, social, cultural, and personal criteria.

Some limitations of the study may be important to consider when interpreting our findings. There is of course the possibility that the participants might have wanted to please the interviewers who, in their world of thinking, might have represented Western midwifery and medicine. That would have induced a bias against one of the main findings in the study—that the “modern” care does not provide the women with a sense of authority. The interviewers were aware of this possible bias, which they tried to avoid. The time for provision of information from women was the period since the most recent childbirth. This may in some cases appear to be a long period of time and could contribute to a certain degree of information bias. However, experience and impressions of childbirth appear to have a lifelong imprint in the minds of women [40–42], and we believe this has not limited the study.

## 5. Conclusions

The study shows the importance to Yemeni women of executing own choice in matters of childbirth. To receive birth support is a primary concern that women have. The strong community ties and dynamic cooperation of women around traditional childbirth could thus be used as a foundation stone for the incorporation of women's demands into professional delivery care. Midwives should receive training in the skills they need to effectively support a woman during childbirth, and cooperation should be sought with the traditional childbirth sector in rendering this training as client oriented as possible. There is a need for more qualitative, in-depth research to gain insight into women's perception of safety and its influence on the choices women make regarding both birth attendance and location of childbirth. This knowledge is important in achieving Safe Motherhood and for the progress towards the Millennium Development Goal 5.

## Figures and Tables

**Table 1:** Location of the previous childbirth (home/clinic or health unit/hospital) among women in the study population by selected factors (*n*: 220).

	Home birth (%)	Clinic birth (%)	Hospital birth (%)	Totals (%)
*Social and demographic factors*				
Women's age				
<25 years	42 (70)	4 (7)	14 (23)	60 (100)
25–35 years	76 (67)	8 (7)	30 (26)	114 (100)
>35 years	33 (72)	2 (4)	11 (24)	46 (100)
*P* value = .952				
Place of residence				
Urban	66 (59)	13 (12)	33 (29)	112 (100)
Rural	85 (79)	1 (1)	22 (20)	108 (100)
*P* value = .0006				
Number of dependents				
2–5	22 (47)	3 (6)	22 (47)	47 (100)
6–9	42 (66)	7 (11)	15 (23)	64 (100)
10–13	54 (78)	4 (6)	11 (16)	69 (100)
>14	33 (83)	0 (0)	7 (17)	40 (100)
*P* value = .001				
Dead children				
Yes	102 (69)	8 (5)	38 (26)	148 (100)
No	49 (68)	6 (8)	17 (24)	72 (100)
*P* value = .691				
Antenatal care				
Yes	93 (61)	12 (8)	47 (31)	152 (100)
No	58 (85)	2 (3)	8 (12)	68 (100)
*P* value = .002				
Distance by walking to location of childbirth				
None (home birth)	153 (100)	0 (0)	0 (0)	153 (100)
<half an hour	0 (0)	11 (58)	8 (42)	19 (100)
half an hour–one hour	0 (0)	1 (14)	6 (86)	7 (100)
one hour–two hours	0 (0)	0 (0)	1 (100)	1 (100)
>two hours	0 (0)	1 (2)	39 (98)	40 (100)
*P* value <.0001				

Literacy and education				
Women's literacy				
Yes	33 (54)	8 (13)	20 (33)	61 (100)
No	118 (74)	6 (4)	35 (22)	159 (100)
*P* value = .005				
Women's literacy and education				
Illiterate	114 (74)	5 (3)	35 (23)	154 (100)
Reads Quran	4 (66)	1 (17)	1 (17)	6 (100)
Primary school	20 (51)	7 (18)	12 (31)	39 (100)
Intermediary school	4 (67)	0 (0)	2 (33)	6 (100)
Secondary school	7 (54)	1 (8)	5 (38)	13 (100)
Higher education	2 (100)	0 (0)	0 (0)	2 (100)
*P* value = .058				
Husbands' literacy and education				
Illiterate	67 (79)	0 (0)	18 (21)	85 (100)
Reads Quran	6 (75)	0 (0)	2 (25)	8 (100)
Primary school	20 (50)	4 (10)	16 (40)	40 (100)
Intermediary school	17 (77)	2 (9)	3 (14)	22 (100)
Secondary school	18 (60)	6 (20)	6 (20)	30 (100)
Higher education	23 (66)	2 (6)	10 (28)	35 (100)
*P* value = .005				

Occupation and income				
Women's employment and wage salary during the last 3 months				
Yes	7 (41)	3 (18)	7 (41)	17 (100)
No	144 (71)	11 (5)	48 (24)	203 (100)
*P* value = .022				
Occupational status of husband				
Farmer/laborer	72 (73)	3 (3)	23 (24)	98 (100)
Government employee	79 (65)	11 (9)	32 (26)	122 (100)
*P* value = .15				
Other family income (land/cattle)				
Yes	43 (88)	0 (0)	6 (12)	49 (100)
No	108 (63)	14 (8)	49 (29)	171 (100)
*P* value = .003				

*Factors related to childbirth*				
Own choice of birth attendance/location of childbirth				
Yes	133 (73)	11 (6)	39 (21)	183 (100)
No	18 (49)	3 (8)	16 (43)	37 (100)
*P* value = .013				
Birth complications (self-rated)				
Yes	25 (40)	5 (8)	32 (52)	62 (100)
No	126 (80)	9 (6)	23 (14)	158 (100)
*P* value <.0001				
Women's authority during childbirth				
Yes	135 (85)	10 (6)	15 (9)	160 (100)
No	16 (27)	4 (7)	40 (66)	60 (100)
*P* value <.0001				
Presence of birth support person(s)				
Yes	128 (92)	6 (4)	5 (4)	139 (100)
No	23 (28)	8 (10)	50 (62)	81 (100)
*P* value <.0001				
Mother-infant proximity following birth (*n* = 217, 3 stillborn children)				
Skin-to-skin	17 (100)	0 (0)	0 (0)	17 (100)
Wrapped/dressed in arms	62 (86)	2 (3)	8 (11)	72 (100)
Separate bed/elsewhere near	55 (68)	8 (10)	18 (22)	81 (100)
Other place in room but out of contact	15 (38)	2 (5)	22 (57)	39 (100)
Separate room/place out of sight	2 (25)	1 (12)	5 (63)	8 (100)
*P* value <.0001				

**Table 2:** Variables with significant explanatory power in multiple logistic regression using previous location of childbirth (home/clinic/hospital) as a dependent variable (*n*: 220).

Explanatory variable	Odds ratio (OR)	Confidence interval (CI) 95%
Women's authority during childbirth	OR 1.26	CI 1.12–1.42
Presence of birth support person(s)	OR 1.36	CI 1.21–1.53
Own choice of birth attendance/location of childbirth	OR 1.14	CI 1.01–1.27
Antenatal care	OR 1.11	CI 1.01–1.22
Family income from land/cattle	OR 1.14	CI 1.03–1.26
Distance to the location of childbirth	OR 1.47	CI 1.41–1.52

Other variables (which did not make independent significant predictions) included in the multiple logistic regression analysis were women's literacy, women's employment and wage salary during the last three months, husbands' literacy and education, number of dependents, birth complications, mother-infant proximity, and urban/rural place of residence.

**Table 3:** Preferred location of a future childbirth (same/different) among women in the study population by selected factors (*n*: 215, five women stated that they would not be pregnant again).

	Same location (%)	Different location (%)	Totals (%)
*Social and demographic factors*			
Women's age			
<25 years	52 (87)	8 (13)	60 (100.0)
25–35 years	97 (87)	15 (13)	112 (100.0)
>35 years	37 (86)	6 (14)	43 (100.0)
*P* value = .995			
Place of residence			
Urban	95 (87)	14 (13)	109 (100)
Rural	91 (86)	15 (14)	106 (100)
*P* value = .779			
Number of dependents			
2–5	39 (85)	7 (15)	46 (100)
6–9	56 (89)	7 (11)	63 (100)
10–13	55 (83)	11 (17)	66 (100)
>14	36 (90)	4 (10)	40 (100)
*P* value = .702			
Dead children			
Yes	63 (88)	9 (12)	72 (100)
No	123 (86)	20 (14)	143 (100)
*P* value = .763			
Antenatal care			
Yes	129 (87)	20 (13)	149 (100)
No	57 (86)	9 (14)	66 (100)
*P* value = .966			
Distance by walking to location of childbirth			
None (home birth)	140 (93)	10 (7)	150 (100)
< half an hour	16 (84)	3 (16)	19 (100)
half an hour–one hour	5 (71)	2 (29)	7 (100)
one hour–two hours	0 (0)	1 (100)	1 (100)
>two hours	25 (66)	13 (34)	38 (100)
*P* value <.0001			

Literacy and education			
Women's literacy			
Yes	53 (87)	8 (13)	61 (100)
No	133 (86)	21 (14)	154 (100)
*P* value = .92			
Women's literacy and education			
Illiterate	127 (85)	22 (15)	149 (100)
Reads Quran	6 (100)	0 (0)	6 (100)
Primary school	33 (85)	6 (15)	39 (100)
Intermediary school	5 (83)	1 (17)	6 (100)
Secondary school	13 (100)	0 (0)	13 (100)
Higher education	2 (100)	0 (0)	2 (100)
*P* value = .602			
Husbands' literacy and education			
Illiterate	70 (86)	11 (14)	81 (100)
Reads Quran	8 (100)	0 (0)	8 (100)
Primary school	30 (77)	9 (23)	39 (100)
Intermediary school	21 (95)	1 (5)	22 (100)
Secondary school	26 (87)	4 (13)	30 (100)
Higher education	31 (89)	4 (11)	35 (100)
*P* value = .311			

Occupation and income			
Women's employment and wage salary during the last 3 months			
Yes	13 (76)	4 (24)	17 (100)
No	173 (87)	25 (13)	198 (100)
*P* value = .207			
Occupational status of husband			
Farmer/laborer	79 (85)	14 (15)	93 (100)
Government employee	107 (88)	15 (12)	122 (100)
*P* value = .557			
Other family income (land/cattle)			
Yes	43 (91)	4 (9)	47 (100)
No	143 (85)	25 (15)	168 (100)
*P* value = .258			

*Factors related to the previous childbirth*			
Location of childbirth			
Home	140 (95)	8 (5)	148 (100)
Institution	46 (69)	21 (31)	67 (100)
*P* value <.0001			
Own choice of birth attendance/location of childbirth			
Yes	165 (92)	14 (8)	179 (100)
No	21 (58)	15 (42)	36 (100)
*P* value <.0001			
Birth complications (self-rated)			
Yes	42 (70)	18 (30)	60 (100)
No	144 (93)	11 (7)	155 (100)
*P* value <.0001			
Women's authority during childbirth			
Yes	147 (94)	10 (6)	157 (100)
No	39 (67)	19 (33)	58 (100)
*P* value <.0001			
Presence of birth support person(s)			
Yes	131 (96)	6 (4)	137 (100)
No	55 (70)	23 (30)	78 (100)
*P* value <.0001			
Mother-infant proximity following birth (*n* = 212, 3 stillborn children)			
Skin-to-skin	14 (93)	1 (7)	15 (100)
Wrapped/dressed in arms	66 (92)	6 (8)	72 (100)
Separate bed/elsewhere near	69 (87)	10 (13)	79 (100)
Other place in room but out of contact	29 (76)	9 (24)	38 (100)
Separate room/place out of sight	5 (63)	3 (37)	8 (100)
*P* value = .05			

**Table 4:** Variables with significant explanatory power in multiple logistic regression using prospective location of childbirth (same/different) as a dependent variable (*n*: 215).

Explanatory variable	Odds ratio (OR)	Confidence interval (CI) 95%
Own choice of birth attendance/location of childbirth	OR 7.26	CI 2.66–19.84
Presence of birth support person(s)	OR 5.05	CI 1.4–18.24
Birth complications	OR 3.5	CI 1.2–10.22

Other variables (which did not make independent significant predictions) included in the multiple logistic regression analysis were: women's authority during childbirth and distance by walking to the location of childbirth.
